# Bibliometric analysis of NMDA receptors: 2015–2024

**DOI:** 10.3389/fphar.2025.1614831

**Published:** 2025-06-06

**Authors:** Yunsheng Liu, Yanyan Jia, Zengwei Kou

**Affiliations:** ^1^ Cancer Center, Shenzhen Hospital (Futian) of Guangzhou University of Chinese Medicine, Shenzhen, China; ^2^ Department of Neurosurgery, Institute of Translational Medicine, Shenzhen Second People’s Hospital, The First Affiliated Hospital of Shenzhen University Health Science Center, Shenzhen, China; ^3^ ENT Institute and Otorhinolaryngology Department of Eye & ENT Hospital, State Key Laboratory of Medical Neurobiology and MOE Frontiers Center for Brain Science, Fudan University, Shanghai, China; ^4^ Department of Laboratory Medicine and Pathobiology, Temerty Faculty of Medicine, University of Toronto, Toronto, ON, Canada

**Keywords:** glutamate receptor, brain, citespace, VOSviewer, bibliometric study

## Abstract

NMDA receptors, a subtype of ionotropic glutamate receptors, play pivotal roles in the brain by mediating synaptic signal transduction, facilitating intercellular communication, and shaping neural circuits, thereby serving as molecular switches for learning and memory. Since its discovery and isolation in the 1960s, NMDA receptors have remained a focal point of research in neuroscience. The past decade has witnessed a large number of high-quality studies on the biophysical properties, three-dimensional structure, and pathophysiological functions of NMDA receptors. In this study, we employed bibliometric methods to analyze publications from 2015 to 2024, visualizing research hotspots, trends, and key milestones of NMDA receptors. Additionally, we also identified the leading researchers, institutions, and countries that contributed to this area. Our findings provide a comprehensive overview of recent NMDA receptor research, which will help readers understand the trends and influence in this field.

## 1 Introduction

NMDA receptors (NMDARs) are widely expressed excitatory ion channel receptors in the brain (Traynelis et al., 2010; Xiong et al., 2025). They typically forms heterotetrameric complexes composed of two GluN1 subunits and two identical or different GluN2 or GluN3A subunits (Hansen et al., 2018; Liu et al., 2024). Their activation requires both binding to agonists, glycine and glutamate, and the depolarization to relieve Mg^2+^ block (Reiner and Levitz, 2018). Owing to this dual requirement for activation, NMDARs are often referred to as “coincidence detectors”.

NMDARs are involved in nearly all aspects of neuronal life, including cell development, fate determination, and transdifferentiation. Given their central role, dysregulation of NMDAR expression, function, or cellular distribution is implicated in a range of severe neurological and psychiatric disorders (Parsons and Raymond, 2014), such as ischemic stroke (Micu et al., 2006), traumatic brain injury (Gabrieli et al., 2021), and autoimmune encephalitis (Zhou et al., 2022; Michalski et al., 2024; Wang et al., 2024). Furthermore, the hyperactivation of NMDARs in specific brain regions has been linked to depression and mood disorders (Chou et al., 2024).

Research on NMDARs has long been a focal point in neurobiology. Early studies have focused on functional aspects, utilizing techniques such as patch-clamp electrophysiology and calcium imaging to explore the biophysical properties of these receptors. Subsequent advances in genetic tools have enabled investigations of the expression and distribution of NMDARs.

Over the past decade, the field has witnessed an explosion of research driven by both technological progress and a sustained scientific interest in NMDARs. For instance, single-cell sequencing has unveiled detailed spatiotemporal expression patterns across diverse cell types, while breakthroughs in structural biology have revealed high-resolution three-dimensional structures of NMDARs, significantly enhancing our mechanistic understanding. Notably, many of these studies have employed multi-method, multi-level strategies to address complex scientific questions, making it increasingly difficult to categorize or synthesize the literature in a straightforward manner.

This complexity highlights the urgent need for a comprehensive study that systematically summarizes, analyzes, and classifies recent research, particularly from the past 10 years, to identify key themes, emerging trends, pivotal contributors, institutions, and countries shaping the field. Motivated by this need, we conducted the present study.

In this study, we employed bibliometric methods to analyze publications published during 2015–2024 related to NMDARs retrieved from the Web of Science and PubMed databases. Using specialized software tools, such as CiteSpace (Chen, 2005) and VOSviewer (Waltman et al., 2010), we conducted a comprehensive visualization and in-depth analysis of the research landscape. This approach allowed us to identify research hotspots, key contributors, and leading institutions in the field of NMDAR research.

## 2 Methods

### 2.1 Data acquisition and analysis

We used the Web of Science and PubMed databases for data collection. Web of Science is a premier research platform that provides comprehensive information across the sciences, social sciences, arts, and humanities. It is recognized as an independent global citation database by one of the world’s most trusted publishers. PubMed, on the other hand, is extensively accessed in the medical field and is among the most frequently used databases by healthcare professionals and researchers.

For the Web of Science, we employed the following search query:

TS = (NMDA receptor OR N-Methyl-D-aspartate receptor OR GluN* OR NR1 OR NR2 OR NR3) AND LA = English AND PY = (2015–2024).**

For PubMed, the search query used was:

(“NMDA receptor” [Title/Abstract] OR “N-Methyl-D-Aspartate receptor” [Title/Abstract] OR “GluN” [Title/Abstract] OR “NR1” [Title/Abstract] OR “NR2” [Title/Abstract] OR “NR3” [Title/Abstract]) AND (“2015”[Date-Publication]: “2024”[Date-Publication]) AND English [Language].

The retrieved results were preprocessed and converted into an appropriate format using CiteSpace. After merging the datasets, the data were imported into CiteSpace (version 6.4. R1) and VOSviewer (version 1.6.20) for in-depth bibliometric analysis, enabling the visualization and exploration of research trends, hotspots, and key contributions in the field of NMDARs studies.

In our workflow, we primarily adhered to the default settings for each software. When the volume of data displayed exceeded the manageable level, we applied a threshold to limit the number of displayed items to 500. Additionally, during the analysis of research institutions, we observed that both CiteSpace and VOSviewer treated sub-level academic units (e.g., Department of Neuroscience, Department of Neurology) as independent entities. To ensure accuracy, we excluded institutions with fewer than five citations in VOSviewer and performed manual verification by three independent researchers.

All statistical analyses in this study were conducted using the built-in algorithms of the aforementioned software tools without any manual modifications.

## 3 Results

### 3.1 Overall scale analysis of the data: annual and cumulative publication volume

**FIGURE 1:**
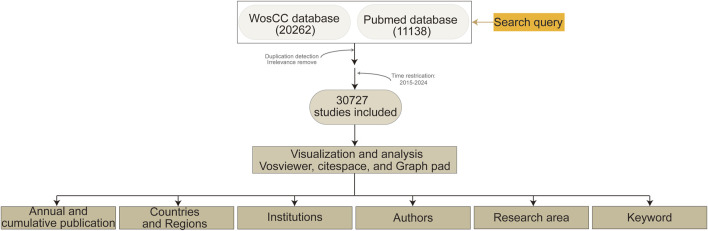
Flow chart of literature search and screening.

A total of 20,262 articles were retrieved from the Web of Science Core Citation Database and 11,138 articles were obtained from PubMed (search conducted on 18 April 2025). We used CiteSpace to reformat the PubMed records to align them with the Web of Science format and subsequently merged the two datasets. After applying duplicate removal and time-based filtering to eliminate potential redundancies and publications outside of the target period, 30,727 unique records were retained. CiteSpace recognized 30,693 records (Figure 1).

Over the past decade (2015–2024), the annual publication volume of NMDARs has remained relatively stable, fluctuating between 2,500 and 3,500 articles per year. However, a gradual downward trend is apparent, with an overall annual growth rate of −3.28%. Notably, a peak in the publication output occurred in 2021, which may be associated with the COVID-19 pandemic. This is supported by a slightly higher proportion of review articles published in 2021 (16.7%) than the overall average.

**FIGURE 2:**
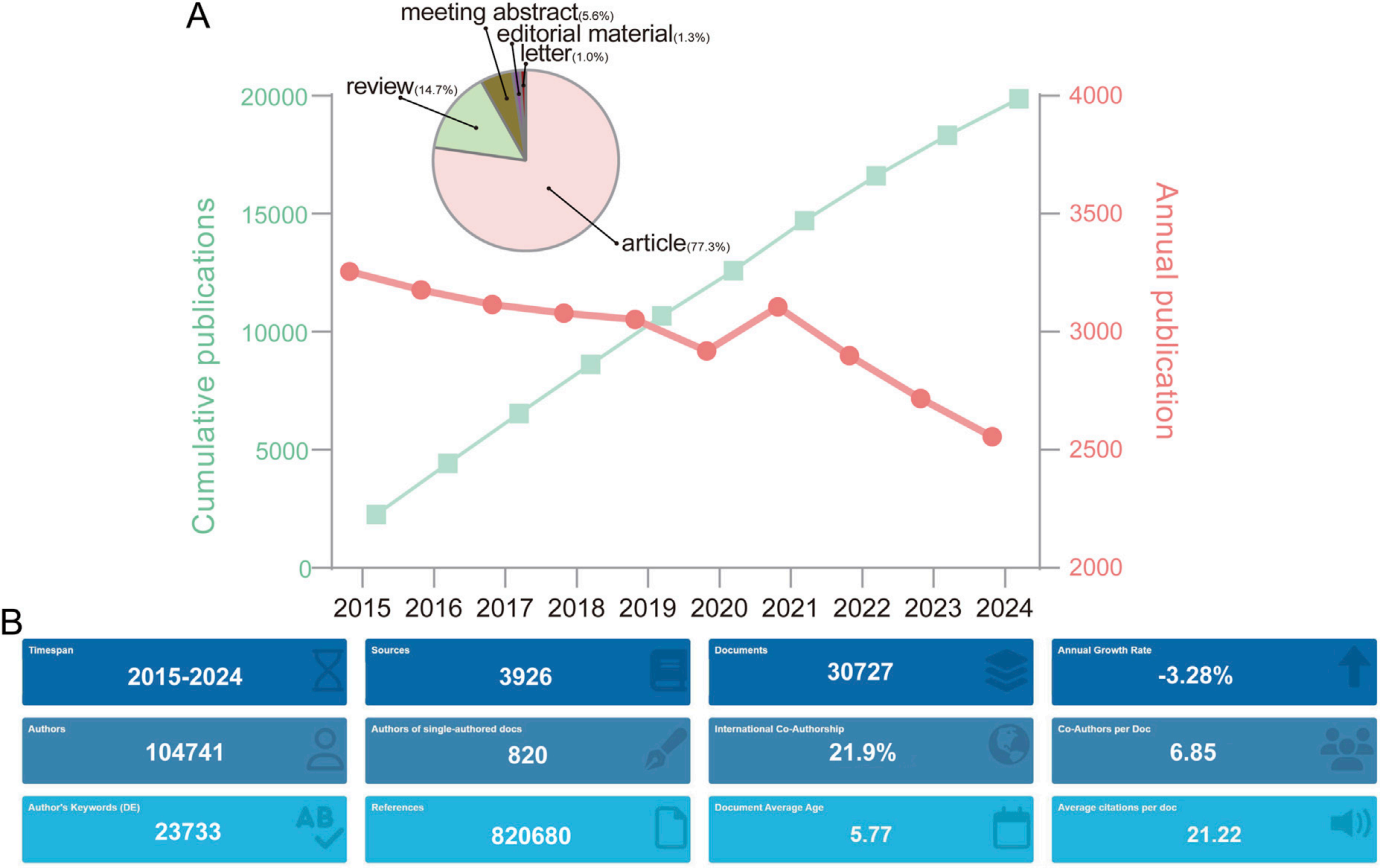
**(A)** Temporal trends in NMDA receptor research output with cumulative publication counts. The inset pie charts depict proportional distribution of publication types. **(B)** Bibliometric summary of publication characteristics.

Among the 30,727 publications analyzed, 77.3% are original research articles, 14.7% are reviews, 5.6% are meeting abstracts, 1.3% are editorial materials, and 1.0% are letters (Figure 2). These articles were published across 3,926 unique sources, with an average of 21.22 citations per document. On an average, each article was authored by 6.85 individuals, although 820 articles were single-authored. Furthermore, international collaboration was notable, with 21.9% of publications resulting from cross-country cooperation.

### 3.2 Countries, institutions, and individuals with the highest publication output

**FIGURE 3:**
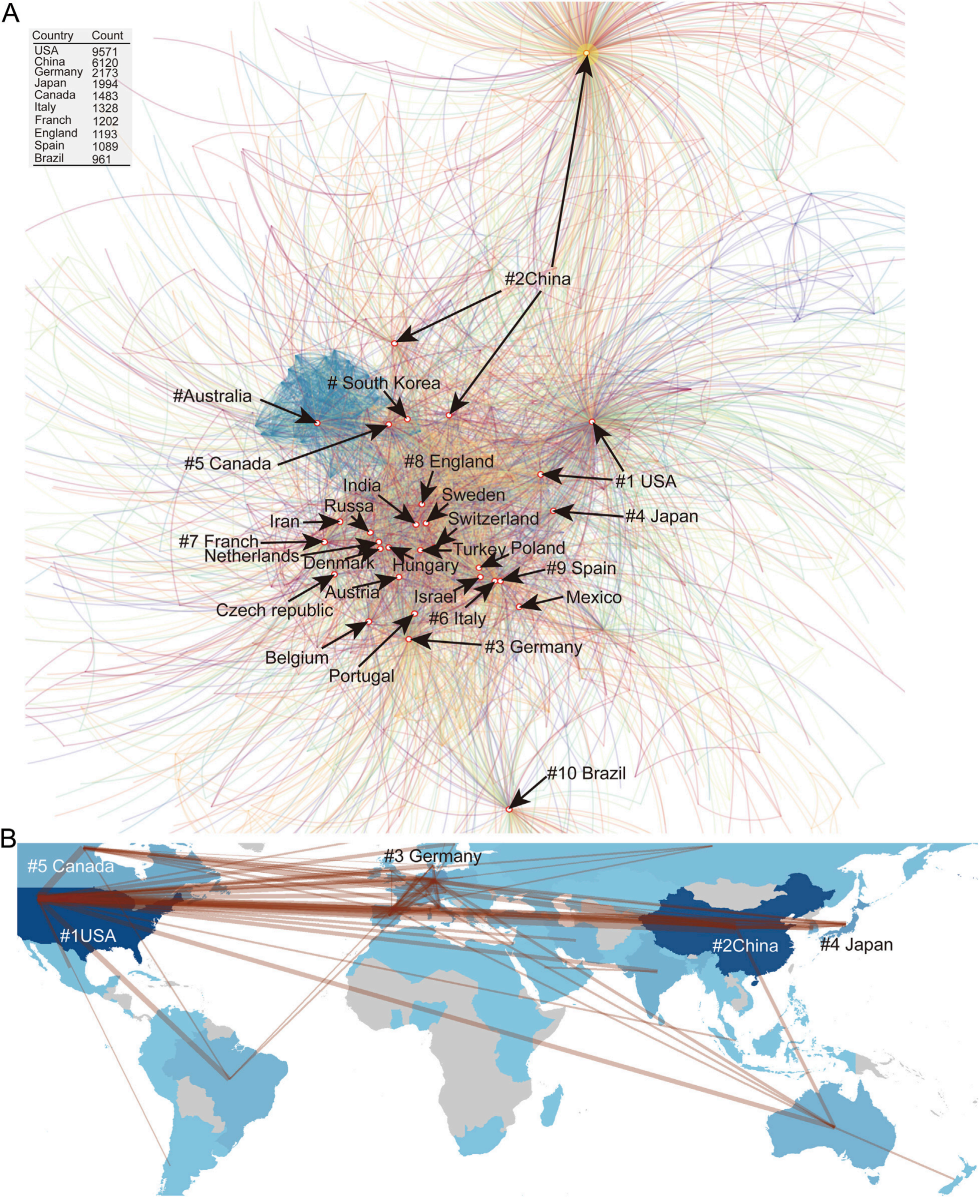
**(A)** Collaborative network mapping of countries engaged in NMDA receptor research. Inset table ranks the top 10 contributing nations by publication count. **(B)** Geospatial visualization of international research cooperation patterns.

We further analyzed the countries, institutions, and individuals with the highest number of publications. The United States leads with 9,571 publications, which is far ahead of other countries. The second to fifth positions are occupied by China (6,120), Germany (2,173), Japan (1,994), and Canada (1,483). The following countries are Italy (1,328), France (1,202), England (1,193), Spain (1,089), and Brazil (961). It is important to note that variations in country names were standardized during data processing (Figure 3). Because of differences in the traditional labelling of country names, abbreviated shorthand, and for possible historical reasons, China has three cores detected, and the United States is detected by two cores.

Certain patterns of international collaborations have emerged. European countries exhibit strong cooperative relationships, particularly between Denmark, the Netherlands, and Hungary, as well as Italy and Spain. Therefore, Europe forms the largest core, including India, Iran, and Israel as non-European participants. Surrounding this core are Japan, South Korea, and Canada, which are closely linked. Additionally, two relatively independent cores exist: Australia and the United States of America. The USA forms a separate core due to variations in how the country’s name is written (full name vs abbreviation). Interestingly, China has three distinct cores, two of which are relatively close to the US-Europe core, while the third, a much larger core, is highly independent and distant from the US-Europe network. Brazil forms an independent core in the network (Figure 3).

**FIGURE 4:**
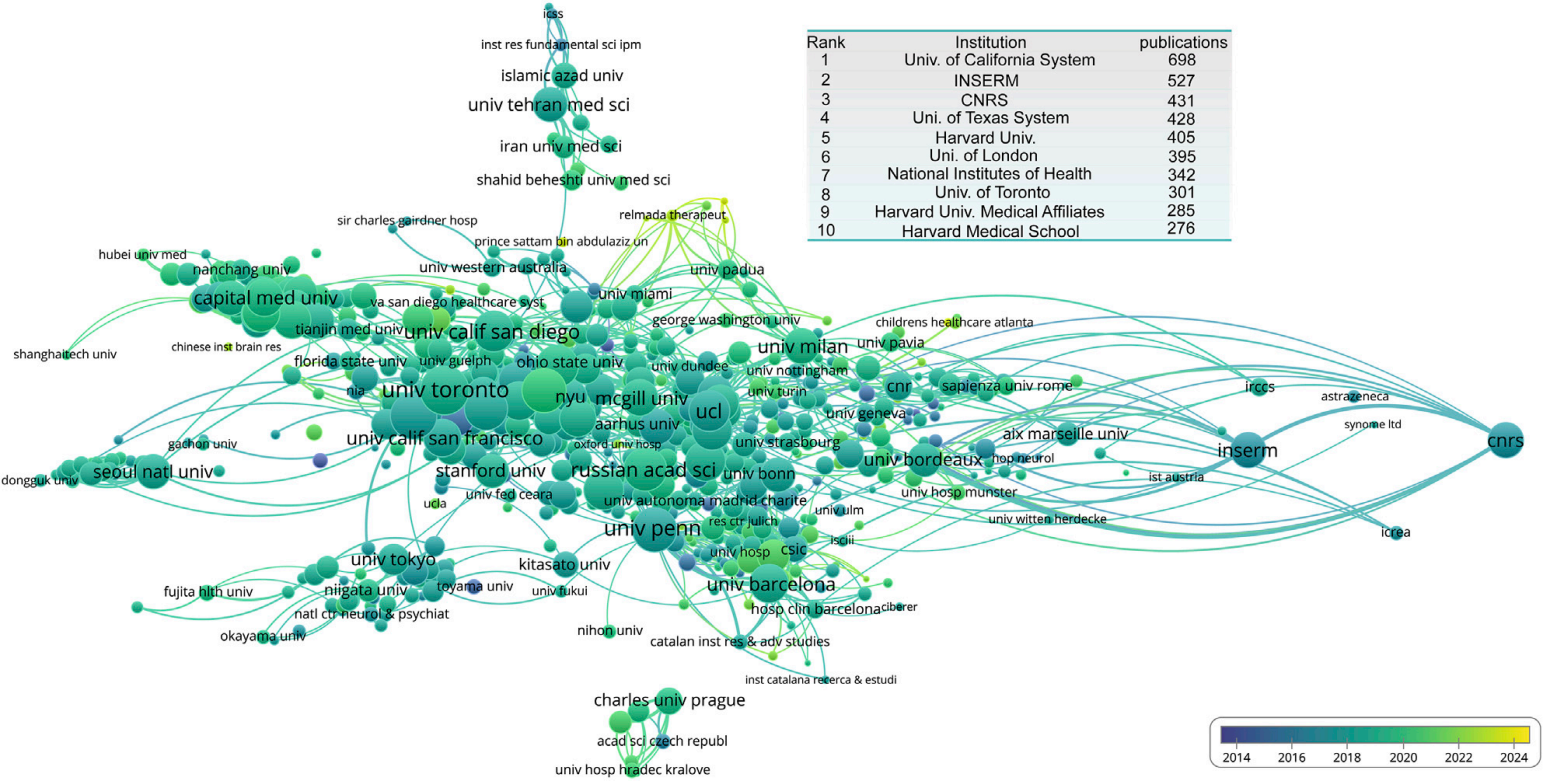
Institutional collaboration network in NMDA receptor research. The inset table lists the top 10 productive institutions based on publication output.

We also analyzed institutions with the highest publication output. The University of California system ranks first with 698 publications, followed by the Institute National de la Santé et de la Recherche Médicale (INSERM) (527), and the Centre National de la Recherche Scientifique (CNRS) (431). From the institutional collaboration and citation network, we observed a research hub centered around the University of California system, with close ties to the University of Toronto and other institutions. Notably, INSERM and CNRS have formed relatively independent research hubs. Beyond this major research core, several smaller hubs are present, including Charles University in Prague, which form a distinct research cluster. The University of Tokyo serves as the center of the Japanese research hub. Seoul National University, representing the South Korean research network. Capital Medical University, leading a Chinese research cluster. The Tehran University of Medical Sciences formed an Iranian research hub (Figure 4).

### 3.3 Publication trends: leading journals and most prolific authors

**FIGURE 5:**
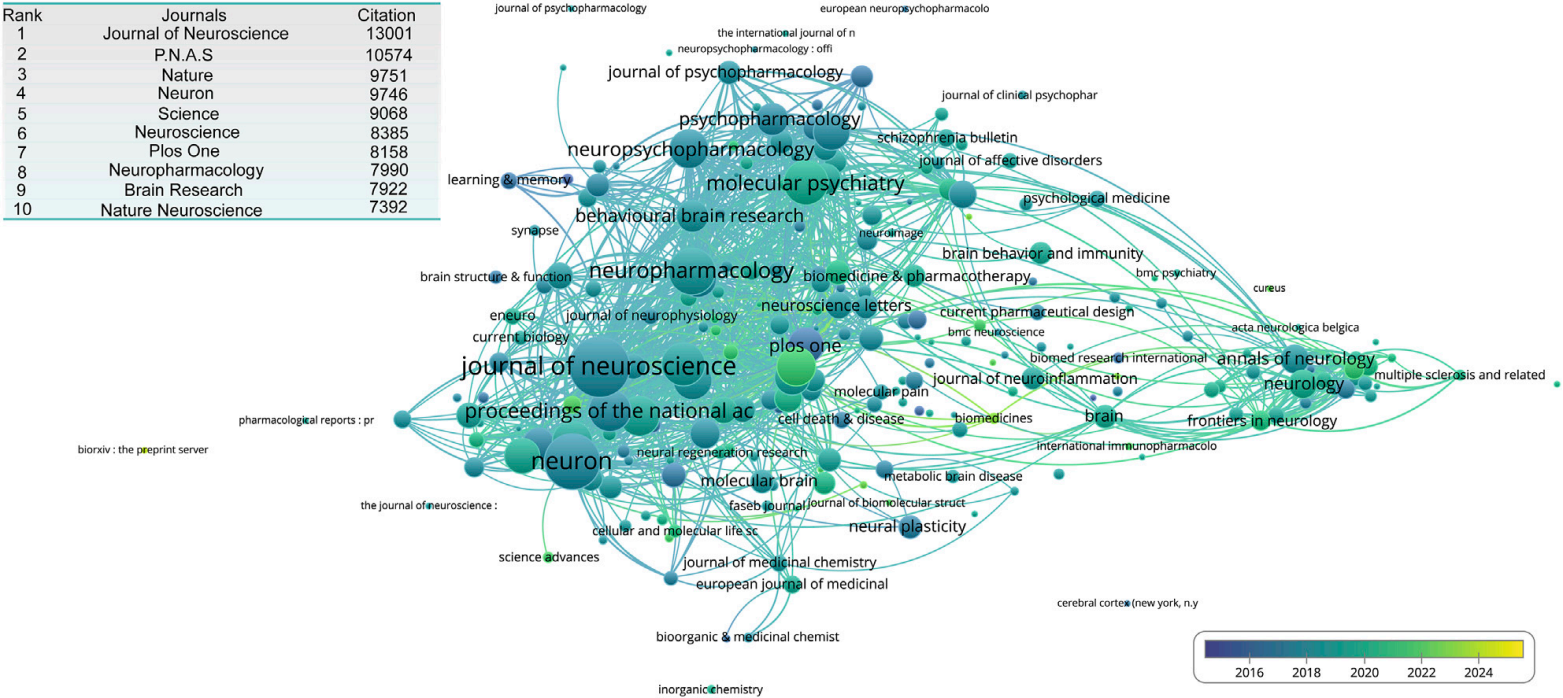
The overlay network of journal engaged in NMDA receptor research. The inserted table shows the top 10 journal contributed to publication on NMDA receptor research.

To evaluate the impact of publications, we analyzed citation counts and visualized the citation distribution. The Journal of Neuroscience had the highest total citation count, reaching 13,001. This was followed by Proceedings of the National Academy of Sciences (PNAS) with 10,574 citations. Other high-impact journals included Nature, Neuron, and Science, indicating their strong influence on the field (Figure 5).

Two major journal clusters emerged from our analysis: One is basic Research Cluster: Centered around journals such as Journal of Neuroscience, Neuron, Neuropharmacology, and Molecular Psychiatry, which primarily represent fundamental neuroscience research. The other one is the Clinical Research Cluster: Led by Neurology and Frontiers in Neurology, which focuses on clinically relevant studies. In addition to these primary clusters, several independent subclusters were identified. For example, the Brain and Journal of Neuroinflammation formed a distinct core, bridging both basic and clinical research. One challenge in clustering was inconsistent journal-naming conventions, which led to duplicate representations. For instance, the Journal of Neuroscience appeared under variations, such as the Journal of Neuroscience, while Cerebral Cortex was listed as Cerebral Cortex (New York, N.Y.) (Figure 5).

Over time, new journals have gained prominence within these clusters: In the Basic Research Cluster, emerging journals include International Journal of Molecular Sciences, Cells, Frontiers in Pharmacology, Molecular Psychiatry, Biomolecules. In the Clinical Research Cluster, new influential journals include Frontiers in Neurology, Frontiers in Immunology, and Neurology Neuroimmunology and Neuroinflammation. Journals bridging both clinical and basic research have also gained traction, with Biomedicines and Brain Sciences standing out as key contributors (Figure 5).

Next, we examined the top 20 most cited and co-occurring publications. These articles were published in 14 prestigious journals, including Nature and The Lancet Neurology. The most highly cited publication was a review article by Graus F et al., published in 2016 in The Lancet Neurology, titled “A clinical approach to diagnosis of autoimmune encephalitis.” This study focused on the relationship between NMDAR targeting and autoimmune encephalitis (AE). Other top-ranking articles related to AE include those ranked third, fifth, 9th to 12th, 15th, and 19th.The second most cited article was a 2016 research paper published in Nature, titled “NMDAR inhibition-independent antidepressant actions of ketamine metabolites.” This study investigated the role of NMDARs in depression. Additional articles focusing on depression included those ranked sixth, seventh, 10th, 14th, and 17th. Moreover, the article ranked 13th to explore the involvement of NMDARs in Alzheimer’s disease (AD). Overall, most of the top 20 articles were disease-oriented, highlighting the clinical relevance of NMDAR research.

Notably, two of the top 20 publications were structural biology studies, elucidating the binding mechanisms of MK801 and ifenprodil to NMDARs and their underlying structural basis (Table 1).

**TABLE 1:** The top 20 of the most cited and occurred publications.

Rank	Freq	Author	Year	Citation	Journal	Type	DOI
1	497	Graus F	2016	4074	Lancet Neurol	Review	10.1016/S1474-4422(15)00401-9
2	301	Zanos P	2016	1626	Nature	Article	10.1038/nature17998
3	297	Dalmau J	2019	826	Lancet Neurol	Review	10.1016/S1474-4422(19)30244-3
4	220	Hansen KB	2018	589	J Gen Physiol	Review	10.1085/jgp.201812032
5	211	Dalmau J	2018	1206	New Neg J Med	Review	10.1056/NEJMra1708712
6	154	Zanos P	2018	1211	Pharmacol Rev	Review	10.1124/pr.117.015198
7	153	Zanos P	2018	1068	Mol Psychiatr	Review	10.1038/mp.2017.255
8	143	Hansen KB	2021	490	Pharmacol Rev	Review	10.1124/pharmrev.120.000131
9	135	Armangue T	2018	631	Lancet Neurol	Article	10.1016/S1474-4422(18)30244-8
10	129	Duman RS	2016	1651	Nat Med	Review	10.1038/nm.4050
11	128	Planagum J	2015	507	Brain	Article	10.1093/brain/awu310
12	109	Balu R	2019	283	Neurology	Article	10.1212/WNL.0000000000006783
13	109	Liu JP	2019	537	Front Neurosci	Review	10.3389/fnins.2019.00043
14	108	Gerhard DM	2020	295	Jclin Invest	Article	10.1172/JCI130808
15	107	Dubey D	2018	772	Ann Neurol	Article	10.1002/ana.25131
16	105	Diering GH	2018	847	Neuron	Review	10.1016/j.neuron.2018.10.018
17	102	Yang Y	2018	955	Nature	Article	10.1038/nature25509
18	99	Song XQ	2018	280	Nature	Letter	10.1038/s41586-018-0039-9
19	94	Dalmau J	2017	635	Physiol Rev	Review	10.1152/physrev.00010.2016
20	108	Tajima N	2016	250	Natrure	Article	10.1038/nature17679

**FIGURE 6:**
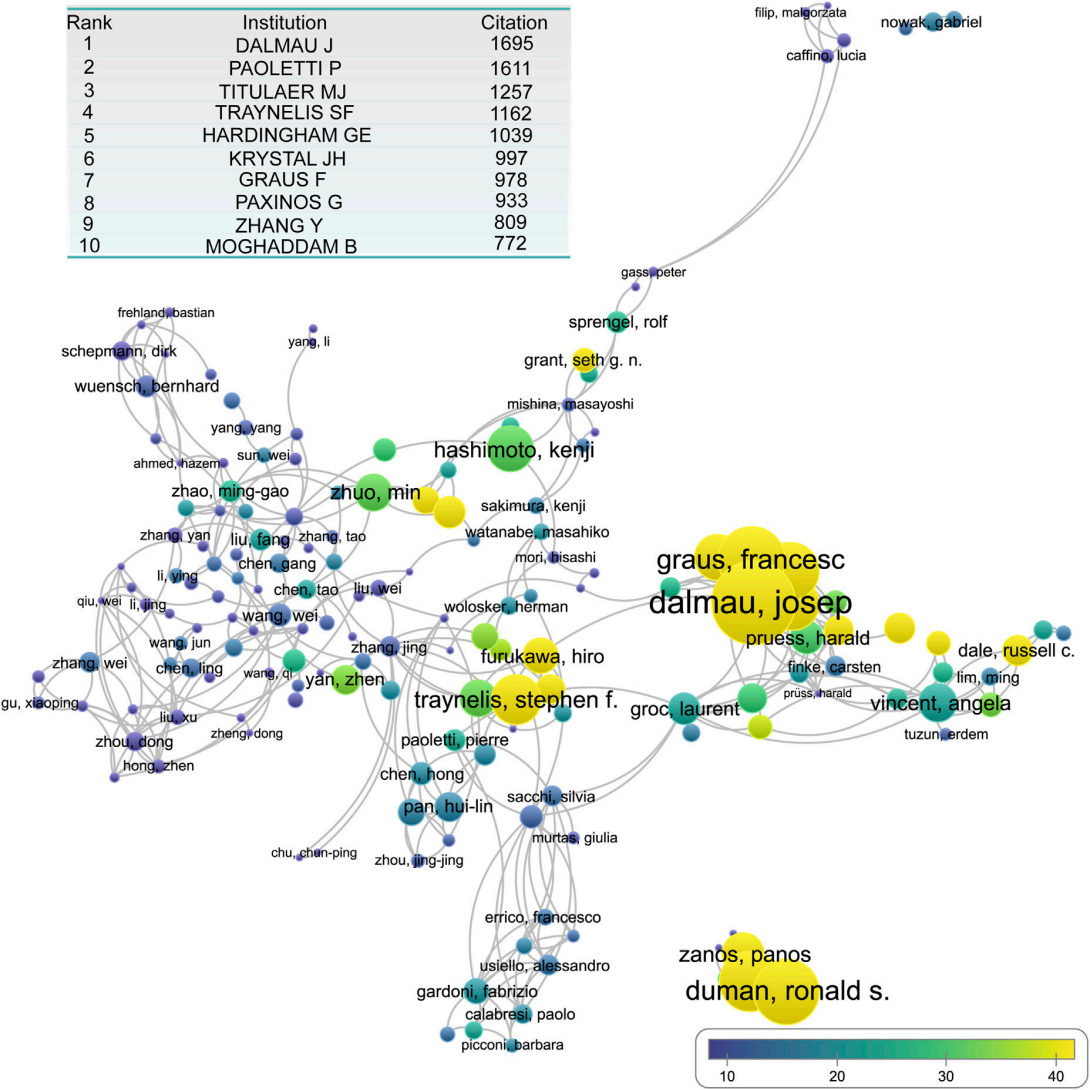
The overlay network of author engaged in NMDA receptor research. The inserted table shows the top 10 author contributed to publication on NMDA receptor research.

To assess the authors’ impact, we analyzed both the total citation counts and average citations per publication. The most cited author was Dalmau Josep, who ranked first in both the total and average citations. Pierre Paoletti ranked second with 1,611 citations, closely following Josep Dalmau (1,695 citations). Maarten J Titulaer secured third place with 1,257 citations. In terms of the average citation count, highly influential authors included: Francesc Graus Ribas, Ronald Duman, Stephen F. Traynelis, Hiro Furukawa, and Kasper B. Hansen (Figure 6).

By analyzing citation networks, we identified distinct author collaboration clusters: Primary Core Cluster: Led by Dalmau Josep and Graus Francesc, which formed the most influential research hub. Secondary Core Cluster: Closely linked to the first, this cluster included Traynelis Stephen F, Hansen Kasper B, and Furukawa Hiro. Independent Research Core: Centered around Duman Ronald, this cluster also included Krystal John H, Zarate Carlos A Jr, and Zanos Panos. Beyond these major clusters, several independent research hubs were identified, with leading researchers such as: Dale Russell C, McKeon Andrew, Collingridge Graham I, Grant Seth G N, Wang Yu Tian, Zhuomin, and Zhenyan. These researchers have contributed significantly to different aspects of NMDAR-related studies, forming a diverse and dynamic research network (Figure 6).

### 3.4 Research hotspots of NMDARs

**FIGURE 7:**
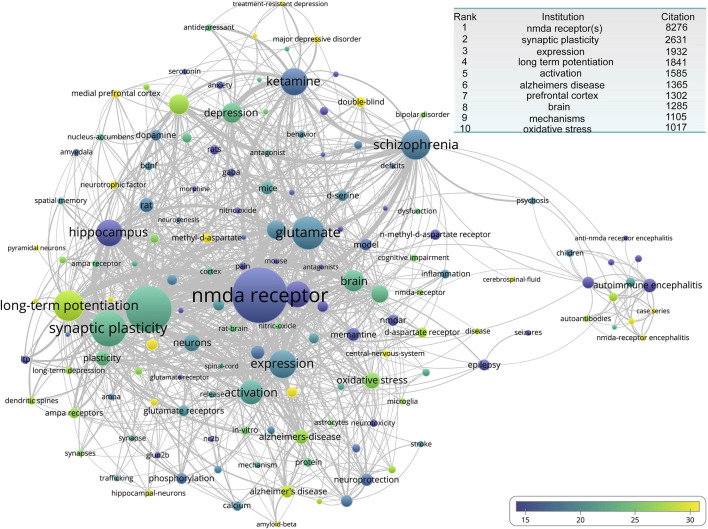
The overlay network of keyword engaged in NMDA receptor research. The inset table presents the top 10 high-frequency keywords associated with NMDA receptor research publications.

We continued the analysis of keywords. Here, we used average citations as the evaluation metric and occurrences as the visualization weights. The most frequent terms were two forms of the NMDA receptor: “NMDA receptor” and “NMDA receptors,” appearing 4,670 and 3,606 times, respectively. These were followed by the primary physiological function of the NMDA receptor, “synaptic plasticity.” The remaining high-frequency keywords are displayed in the insert of the figure. From a clustering perspective, there are two main clusters. One revolves around the “NMDA receptor” and “NMDA receptors,” surrounded by ligands of the NMDA receptor such as “glutamate,” small-molecule drugs like “ketamine”, “D-serine,” and “memantine,” as well as the primary functions of the NMDAR. These include microscopic functions such as “synaptic plasticity”, “oxidative stress,” and “long-term potentiation,” and macroscopic functions like “neuroprotection” and “spatial memory,” along with NMDA-related diseases such as “depression”, “schizophrenia”, “Alzheimer’s disease”, “epilepsy,” and “stroke.” The other cluster centers around “autoimmune encephalitis,” surrounded by terms like “NMDA receptor encephalitis”, “autoantibodies”, and “anti-NMDA receptor encephalitis”. Notably, among these keywords, some of the most frequent ones include “long-term potentiation”, “prefrontal cortex”, “pyramidal neurons”, “methyl-D-aspartate,” and “NMDA-receptor encephalitis” (Figure 7).

### 3.5 Super keyword clustering analysis

**FIGURE 8:**
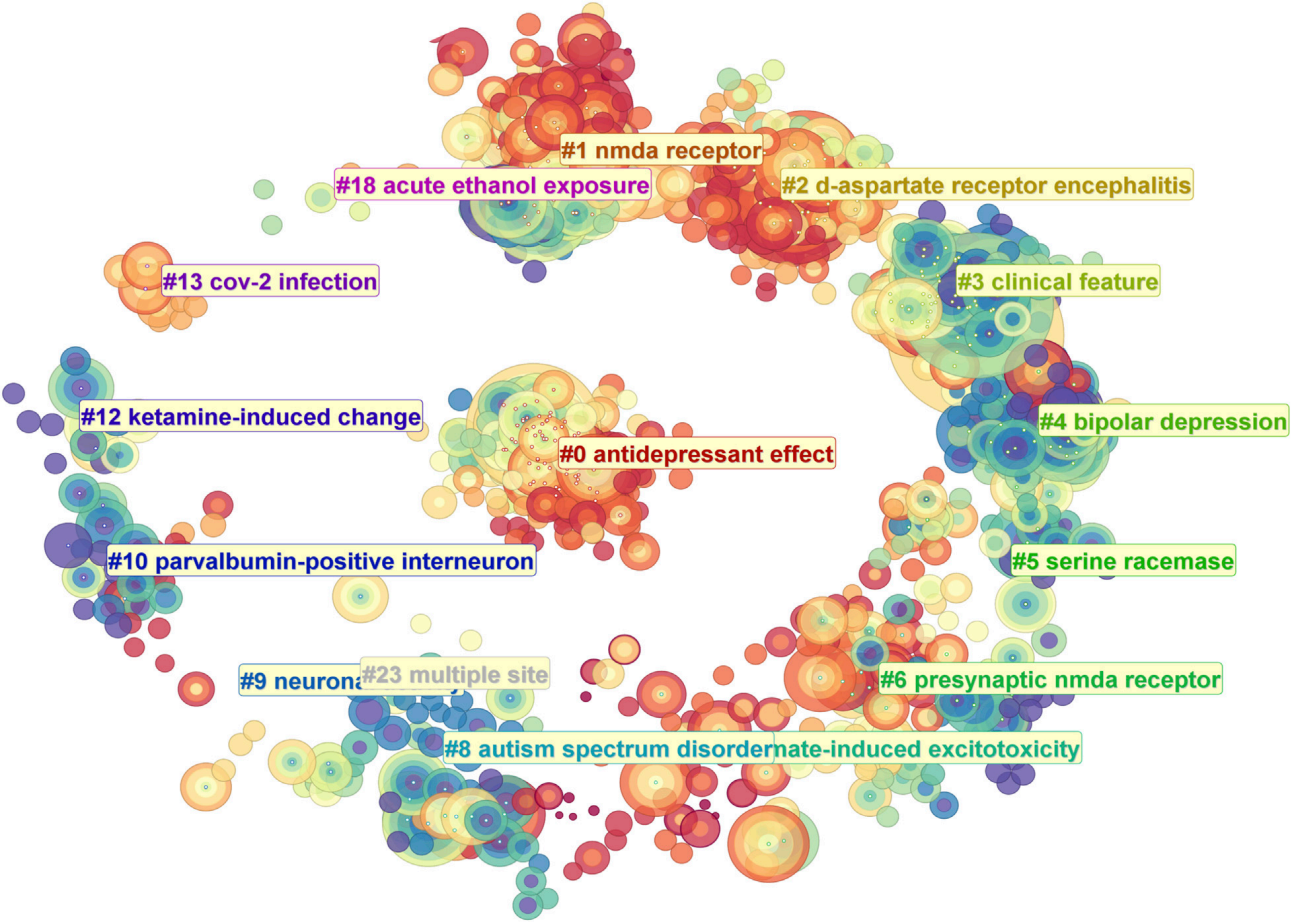
The keyword clustering in NMDA receptor research.

**FIGURE 9:**
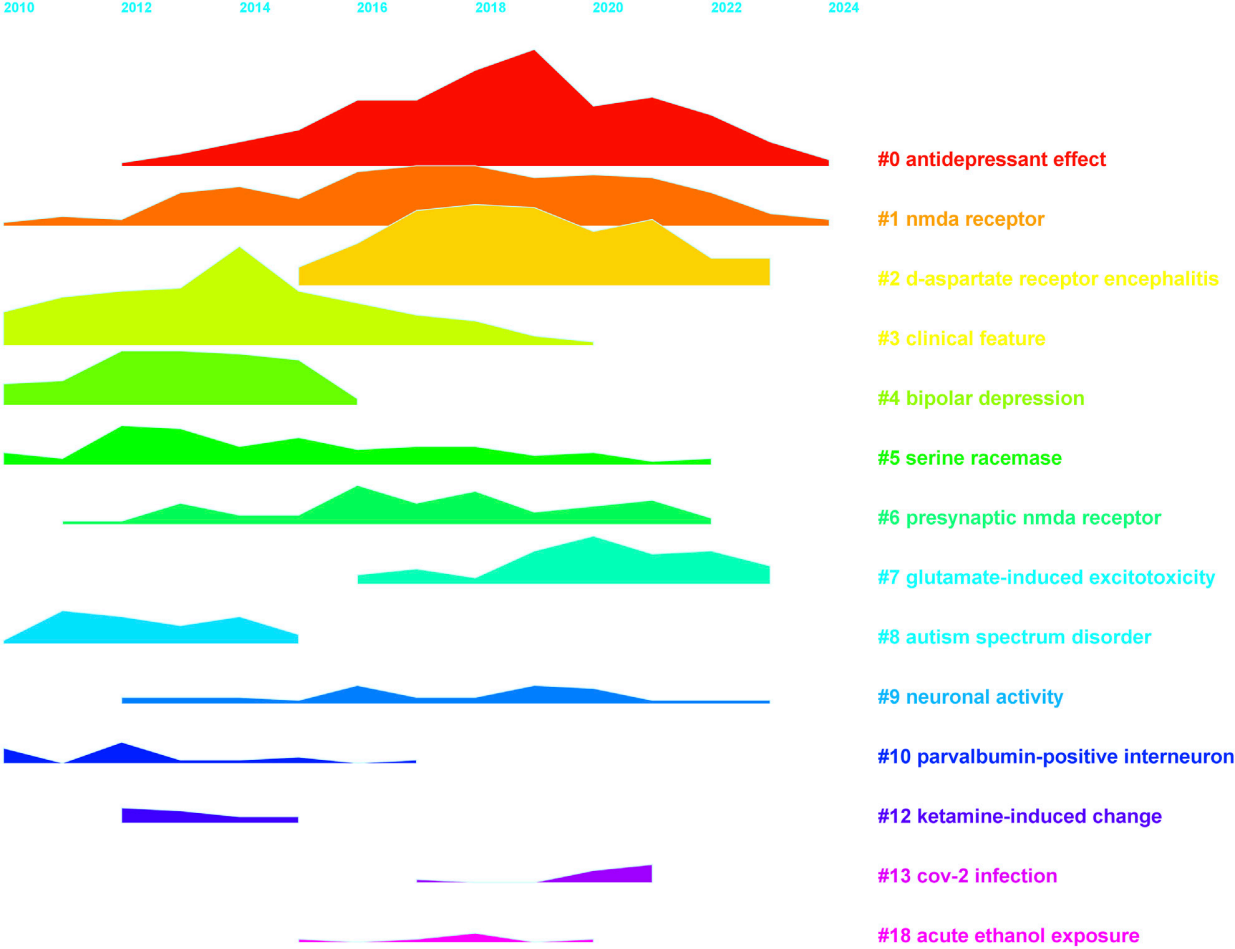
Timeline visualization of keyword cluster evolution, highlighting peak activity periods in NMDA receptor research.

**FIGURE 10:**
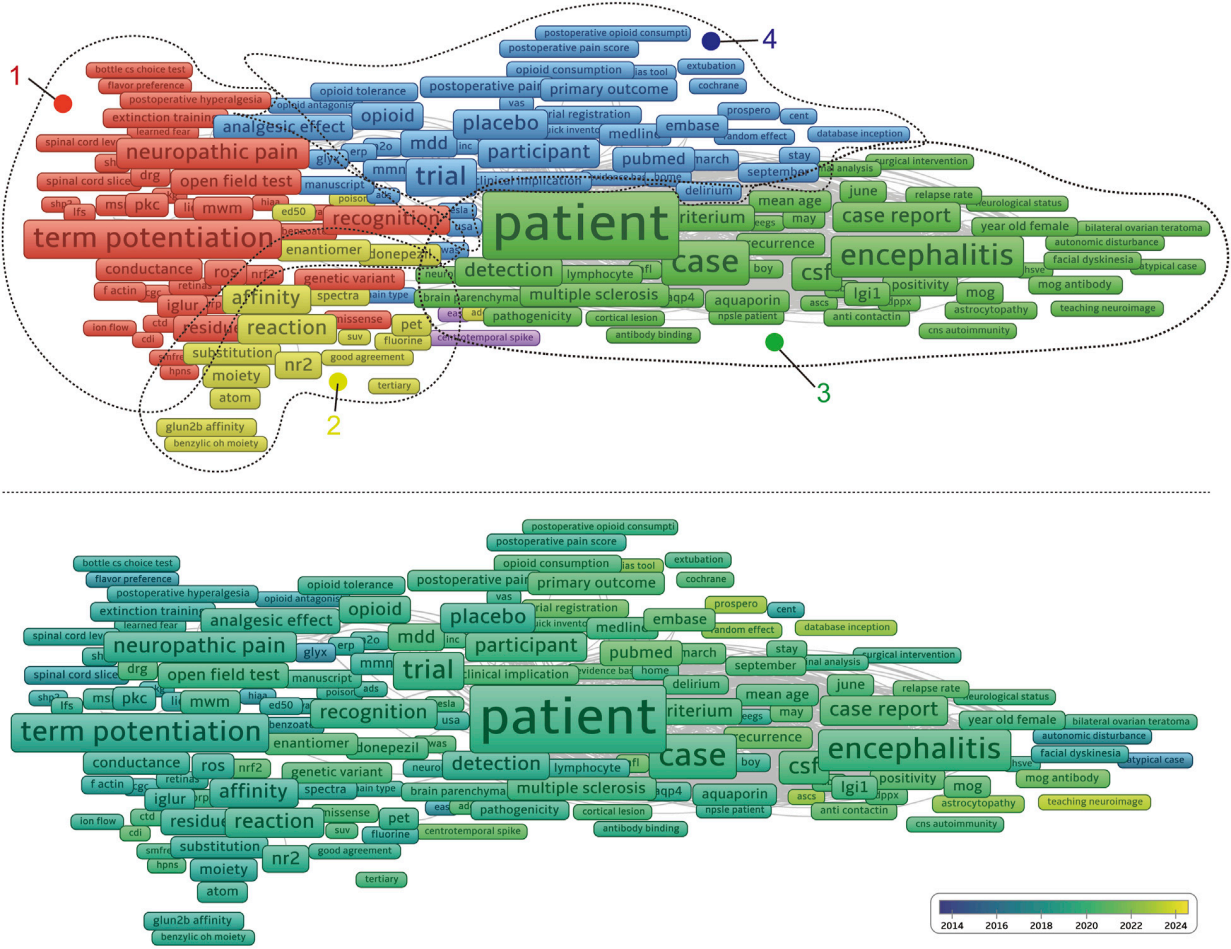
Visualization of NMDA receptor research keyword by occurrence scored by total link strength (top) and publication year (bottom).

Using CiteSpace, we conducted a super keyword clustering analysis across all articles, ranking the top five clusters as follows: “antidepressant effect”, “NMDA receptor”, “D-aspartate receptor encephalitis”, “clinical feature”, and “bipolar depression” (Figure 8). From a temporal perspective, research on the “antidepressant effect” emerged around 2012, followed by a rapid increase in interest, peaking around 2019. Although the research intensity gradually declined thereafter, it remained at a significant level in 2024. In contrast, “d-aspartate receptor encephalitis” began gaining attention around 2015, reached a plateau between 2017 and 2019, and then gradually declined. Notably, there is a minor resurgence in research activity in 2021. The “clinical feature” cluster maintained a high research intensity between 2010 and 2018, peaking in 2014. Other clusters, such as “serine racemase”, “presynaptic NMDA receptor”, and “neuronal activity”, have sustained a high level of research interest from 2014 to 2024. Meanwhile, some topics exhibited distinct temporal trends. For example, “bipolar depression”, “autism spectrum disorder”, and “parvalbumin-positive interneurons” were primarily studied between 2010 and 2014. In contrast, “CoV-2 infection” and “acute ethanol exposure” emerged as research hotspots only after 2015 (Figure 9). We further performed keyword co-clustering analysis using VOS viewer, employing the occurrence frequency as the evaluation metric. The most frequently occurring keyword was “patient”, followed by “encephalitis”, term “potentiation”, and “neuropathic pain”. Given the substantial number of case report-based studies in the literature, “case” and “case report” also appeared with high frequency. Network visualization revealed four major keyword clusters: A basic research-oriented cluster centered around term potentiation. A receptor biophysics-focused cluster centered around the reaction, including key terms, such as GluN2B affinity and tertiary structure. A clinical research-oriented cluster centered around “patient”. A pharmacology-focused cluster centered around the trial. When visualized on a timeline, earlier research (circa 2014) was predominantly focused on fundamental mechanisms, with the terms potentiation and affinity as central themes. Over time, research has gradually shifted toward clinical and pharmacological aspects, with patients, cases, and encephalitis becoming dominant themes in more recent years (Figure 10). This trend highlights a clear transition from basic research toward clinical application.

## 4 Discussion

In this study, we conducted a comprehensive bibliometric analysis of NMDA receptor (NMDAR) research over the past decade (2015–2024). We found that interest in NMDARs has remained consistently high, with a stable annual publication volume ranging from 2,500 to 3,500 papers. Among global institutions, research centers affiliated with the University of California played a pivotal role, positioning the United States as the dominant contributor to this field. Most importantly, we identified several evolving research hotspots and trend shifts in the study of NMDARs over the past decade.

### 4.1 General information

According to our search strategy and the inclusion criteria, a total of 30,727 articles were included in the analysis. Although the number of annual publications remained steady, we observed an overall declining trend in output over the decade. A temporary increase during the COVID-19 pandemic appears to be more of a general phenomenon than specific to this field, and similar trends were observed in our analyses of other receptor families, likely due to reduced access to wet-lab experiments and a redirection of researchers’ efforts toward writing and data analysis during lockdowns.

The long-term decline may be attributed to the maturation of many subfields within NMDAR research. Much of the foundational work, such as subunit cloning, pharmacological characterization, and subtype differentiation, had already reached consensus conclusions by the mid-2010s. While the past decade saw the application of powerful new technologies (e.g., cryo-EM and optogenetics) to the field, these innovations were not sufficient to reverse the broader downtrend. Nevertheless, the field remains robust, with nearly 2,500 publications annually, and we expect another surge of activity when the next major breakthrough emerges.

### 4.2 Top contributing countries, institutions, and journals

The top ten contributing countries accounted for over 88% of all publications, forming a triad of leading regions: North America, Asia, and Europe (Figure 3A). Although Europe contributed fewer papers overall, it demonstrated higher levels of international collaboration. In contrast, countries such as the U.S., Canada, China, and Japan formed more independent research cores with less cross-national cooperation, with the exception of strong collaborative ties between Japan and the U.S (Figure 3B).

China and Brazil showed more self-sufficient research patterns, establishing distinctive focuses and research networks. Notably, the U.S. maintained a consistently high publication volume throughout the decade, forming a central research hub, while China demonstrated rapid growth (data not shown). Germany and Canada maintained stable but moderate outputs.

Institutionally, the U.S. showed a multipolar structure, with the University of California system, the University of Texas system, and Harvard University as the top contributors. These were closely followed by the National Institutes of Health (NIH) (Figure 4). Although China ranked second globally by publication volume, no single Chinese institution entered the top ten, indicating a more decentralized distribution of research efforts.

In terms of citations, The Journal of Neuroscience had the highest impact overall, although interestingly, none of the top 20 most-cited articles were published in that journal. A similar situation was observed for the PNAS (ranked second). Based on our clustering of journal outputs (Figure 5), the literature appears to center on two cores: basic science and clinical research. Although basic research still dominates, in recent years, there has been a gradual shift in focus toward clinically oriented topics.

### 4.3 High impact authors and landmark papers

Among the top-cited authors, a significant number focused on disease-oriented research. Dalmau J, Titulaer MJ, and Graus F made major contributions to the study of anti-NMDAR autoimmune encephalitis (AE). Dalmau’s landmark identification of anti-NMDAR AE in 2005 and subsequent naming of the disease in 2007 established an entirely new research field. Seven of the top 20 most-cited papers addressed various aspects of anti-NMDAR AE (Figure 5; Table 1).

Two other frequently cited researchers-Krystal JH and Moghaddam B, have focused on the link between NMDARs and depression, especially the mechanism and therapeutic applications of ketamine (Figure 6). Their work is reflected in several top-cited papers, including the second- and sixth-most-cited studies (Table 1). Together, studies on anti-NMDAR AE and depression-related mechanisms account for a substantial portion of the most influential research in the field.

### 4.4 Hotspots and Frontiers

Across journal distributions (Figure 5), keyword clustering (Figure 10), top-cited articles (Table 1), and author impact (Figure 6), one clear conclusion emerged: disease-driven research has been the dominant force in NMDAR studies over the past decade. In addition to depression and anti-NMDAR AE, other key topics included neurodegenerative diseases such as Alzheimer’s disease (13th most-cited paper, Table 1) and neurodevelopmental disorders such as autism spectrum disorder (Figure 8).

Basic research on the biochemical and biophysical properties of NMDARs also remained active. Studies on brain-region-specific expression of subtypes, gating mechanisms, and their roles in disease are common, as exemplified by the third and eighth most-cited papers (Table 1). In the domain of structural biology, cryo-EM studies have made substantial contributions, with the 18th and 20th most-cited papers providing high-resolution structures of NMDARs in different conformational states (Table 1).

NMDA receptors have long stood at the crossroads of fundamental neuroscience and clinical neurology. Our bibliometric analysis of studies from 2015 to 2024 shows that the U.S., particularly the University of California system, remains a central force in this research area. Landmark studies by researchers like Dalmau J have shaped the field, especially in relation to anti-NMDAR AE. Meanwhile, interest in NMDARs’ role in depression, especially in relation to ketamine, has surged.

We observed a shift from purely basic research to a more integrated model that balances fundamental discoveries with clinical applications. Disease-driven studies are increasingly defining the field’s direction. We hope that this review provides valuable insight into the evolving landscape of NMDAR research and deepens appreciation for the complex structural and functional roles of receptors in the brain.

In summary, NMDA receptors have long been a focal point of research in neuroscience. By reviewing studies published from 2015 to 2024, we identified the United States as a leading contributor to the field, with the University of California system at the forefront of research institutions. Additionally, Dalmau J and colleagues have published a significant number of high-quality studies in journals such as Journal of Neuroscience. Our analysis of research trends over the past decade reveals a shift from purely basic research to a balance between fundamental and clinical studies, with depression emerging as a primary disease of interest. We hope this review helps readers gain insight into the recent advancements in NMDA receptor research and appreciate the structural and functional unity and diversity of these receptors in the brain.

## Data Availability

The original contributions presented in the study are included in the article/supplementary material, further inquiries can be directed to the corresponding authors.
